# Enhancing medical image privacy in IoT with bit-plane level encryption using chaotic map

**DOI:** 10.3389/fncom.2025.1591972

**Published:** 2025-06-06

**Authors:** Fatima Asiri, Wajdan Al Malwi, Tamara Zhukabayeva, Ibtehal Nafea, Abdullah Aziz, Nadhmi A. Gazem, Abdullah Qayyum

**Affiliations:** ^1^Informatics and Computer Systems Department, College of Computer Science, King Khalid University, Abha, Saudi Arabia; ^2^Department of Information Systems, L.N. Gumilyov Eurasian National University, Astana, Kazakhstan; ^3^College of Computer Science and Engineering, Taibah University, Medina, Saudi Arabia; ^4^High Performance Computing Centre North, Umeå University, Umeå, Västerbotten, Sweden; ^5^Department of Information Systems, College of Business Administration-Yanbu, Taibah University, Medina, Saudi Arabia; ^6^6G Lab, School of Engineering and Informatics University of Sussex, Brighton, United Kingdom

**Keywords:** image encryption, Chen chaotic map, chaos, meaningful encryption, bit-level encryption, IoT

## Abstract

**Introduction:**

Preserving privacy is a critical concern in medical imaging, especially in resource limited settings like smart devices connected to the IoT. To address this, a novel encryption method for medical images that operates at the bit plane level, tailored for IoT environments, is developed.

**Methods:**

The approach initializes by processing the original image through the Secure Hash Algorithm (SHA) to derive the initial conditions for the Chen chaotic map. Using the Chen chaotic system, three random number vectors are generated. The first two vectors are employed to shuffle each bit plane of the plaintext image, rearranging rows and columns. The third vector is used to create a random matrix, which further diffuses the permuted bit planes. Finally, the bit planes are combined to produce the ciphertext image. For further security enhancement, this ciphertext is embedded into a carrier image, resulting in a visually secured output.

**Results:**

To evaluate the effectiveness of our algorithm, various tests are conducted, including correlation coefficient analysis (*C*.*C* < or negative), histogram analysis, key space [(10^90^)^8^] and sensitivity assessments, entropy evaluation [*E*(*S*) > 7.98], and occlusion analysis.

**Conclusion:**

Extensive evaluations have proven that the designed scheme exhibits a high degree of resilience to attacks, making it particularly suitable for small IoT devices with limited processing power and memory.

## 1 Introduction

The Internet of Things (IoT) connects devices and objects via the Internet, whether wirelessly or wired. In recent years, the concept has become increasingly popular as it is used for various purposes, including business development, transportation, education, and communication. The hyper-connectivity created by the IoTs enables individuals and organizations to communicate seamlessly from a distance (Porras et al., 2018). IoT has been widely embraced in a wide range of industries, including e-health, manufacturing, smart cities, agribusiness, and home automation. According to Cisco, Internet-connected gadgets will number approximately 500 billion by 2030 (Aman et al., 2020). As IoT advances exponentially, medical imaging and data have become more widely used, and are therefore need to be secured before being shared.

Medical images have become increasingly important in diagnosing and treating illnesses. The visuals are used directly by doctors during the evaluation and therapy of patients (Ismail et al., 2018). For medical applications, securing the transmission and storage of medical images has become increasingly important due to their containment of private information (Ye and Huang, 2015; Dridi et al., 2016; Al-Haj et al., 2015; Cao et al., 2017; Khan et al., 2018; Hu et al., 2024; Chu et al., 2024; Belazi et al., 2019). A number of academics have therefore focused on developing methods to secure images in IoT applications. The authors in Ye and Huang (2015) utilized logistic and Arnold chaotic maps to design an autoblocking and Electrocardiography (ECG) signal-based medical image encryption scheme. ECG signals and the Wolf algorithm calculates initial conditions for the chaotic system. A key characteristic of this cryptoarchitecture is that it performs autoblocking diffusion only during the encryption phase of the process, in contrast to traditional cryptoarchitectures. A new chaos and neural network-based medical image encryption scheme has been presented in Dridi et al. (2016). Plaintext image pixels are XORed with a generated key. The weight and bias values for neural networks have been computed using the Logistic map. By using this technique, medical images can be made more secure with a simpler algorithm than current ones. Using Strong cryptographic functions with internal symmetric keys and hash codes, the author designed an encryption scheme for medical images that ensures confidentiality, authenticity, and integrity (Al-Haj et al., 2015). With the whirlpool hash function and Galois counter mode, advanced encryption standards are used to secure confidentiality and authenticity, while digital signature algorithms employ elliptic curves to secure integrity and authenticity. The edge map-based medical encryption scheme has been presented in Cao et al. (2017). It consists of three main steps: (a) extraction of bit planes, (b) generating random numbers, and (c) permutations. The source image can be any type of image and distinct edge maps can be produced by varying edge detection approaches and thresholds, depending on the source image type. An Intertwining Logistic map and Deoxyribonucleic acid (DNA) are utilized by Khan et al. to protect medical images (Khan et al., 2018). A DNA sequence is passed through SHA-512 in order to calculate the chaotic system's initial condition. Plaintext pixel correlations are broken down through shuffling. In addition to XORing, an affine transformation is also applied to diffuse the shuffled pixels. A two-round medical encryption scheme is designed by Belazi et al. by combining chaos and DNA (Belazi et al., 2019). During each round, six steps are performed, namely block permutation, pixel substituting, DNA encoding, bit substitution, DNA decoding, and bit diffusion.

As Internet-related technologies continue to grow exponentially, new technologies, energy, or modifications are added daily. Applications and systems that use the Internet of Things benefit greatly from the recent advancements in wireless technology from 1G to 5G (Hasan et al., 2021). In recent years, high-quality medical care has become increasingly important as a result of population growth, urbanization, and the COVID-19 pandemic (Trujillo-Toledo et al., 2023). In medical diagnostics, X-rays, Computer Tomography Scans (CT scans), nuclear medicine imaging, and ultrasounds are modern imaging techniques. Thus, these high-resolution diagnostic images need to be secured before being exchanged. Recently, cyber attacks could make healthy patients appear sick and vice versa. Therefore, cyber-security threats will increase alarmingly in the area of medical image communication. It is therefore increasingly important to have fast and secure cyber-security systems regarding the diagnosis of medical images (Kester et al., 2015). The Internet of Medical Things (IoMT) can provide many advantages to hospitals and healthcare organizations. However, they need to ensure that the right policies and protocols are in place to tackle the security challenges posed by IoMT. Researchers are curious about the potential security and privacy issues associated with this concept, particularly when bandwidth and frequency are high. Therefore, it is essential to design a robust medical image encryption scheme to guarantee the safe and trustworthy transmission and receipt of patients' symptomatic data through IoT. Double permutation techniques are used in Hasan et al. (2021) to design a lightweight, efficient encryption algorithm to protect healthcare images. In this method, plaintext images are broken down into blocks and encrypted. A chaotic encryption technique, based on the Message Queuing Telemetry Transport (MQTT) protocol, is proposed in this research for enhancing security and secrecy when transmitting medical images over the Healthcare Internet of Things (H-IoT) network (Trujillo-Toledo et al., 2023). Initially, chaotic maps are enhanced and applied to encrypt plaintext pixels through diffusion. The designed scheme efficiency is confirmed via a number of tests. The designed embedded medical cryptosystems transmit real-time medical images over the Internet and WiFi, thus enhancing real-time medical image security. Using multiple chaotic maps, the authors propose Multiple Map Chaos Based Image Encryption (MMCBIE) scheme, a novel method that encrypts images in the IoT environment (Jain et al., 2024). Unlike existing schemes, MMCBIE combines multiple chaotic maps, like Henon and 2D Logistic chaotic maps in a unique combination. According to security assessments and cryptanalysis, MMCBIE possesses high-level security properties, making it a superior method of image encryption. Hanchate and Anandan (2024) presented a hybrid scheme that combines Adaptive Elliptic Curve Cryptography (AECC) and Logistic mapping to encrypt medical images for the IoT. As a first step, the image is encrypted using the AECC technique, then again encrypted using the logistic map-based DNA sequence algorithm for greater security. The diffused DNA matrix is then decoded to produce the cipher image. The plain image determines the rules for encoding and decoding DNA as well as the key matrix. Liu et al. (2024) utilize compressive sensing (CS) and chaotic systems to design an encryption scheme for IoT scenarios to ensure security and efficiency. A chaotic laser system generates Masuemet matrices with complex phase space. The measurement matrices are further enhanced through the use of cyclic matrix methods. The image reconstruction quality is further improved using segmented linear thresholding. Further, large images are compressed block-wise in order to reduce storage space and improve reconstruction efficiency. The authors in Nadhan and Jacob (2024) investigated how a cryptography-based network might be able to encode medical images, as well as how deep learning could be used to ensure that the images are transmitted safely. Various image representations have been mapped using the ResNet-50 architecture. As a result of the extensive empirical setup and the security analysis, the suggested method is likely to provide unprecedented levels of security. An IWT-based DNA encoding scheme is proposed to encrypt medical images within the Healthcare IoT (Lai and Hua, 2025). Random sequences were generated using a 3D hyperchaotic map. In addition to IWT, a novel diffusion algorithm masks critical information by generating approximation components. Bit-level permutations further enhance encryption complexity. The scheme further uses the DNA shuffle technique and encrypts the permuted images using a DNA-encoding technique to enhance security.

Most traditional image encryption algorithms convert plain images into noise-like ciphers, making them easily detectable and vulnerable to attack during transmission or storage. Visual security should be considered when designing an image encryption method to avoid hackers' attention. Therefore, to avoid the eavesdropper's attention, meaningful image encryption algorithms must be developed that may generate visually meaningful ciphertext images. Image encryption algorithms that provide a visual sense of meaning have attracted considerable research attention (Khan et al., 2024, 2020; Gan et al., 2024; Sathananthavathi et al., 2024; Zhang et al., 2024). A bit plane image encryption scheme was designed by Khan et al. (2024) using hash function and chaos theory. A SHA-512 hash algorithm is used to compute the key for the chaotic map. The chaotic random vectors are used to shuffle the plaintext image pixels row- and column-wise, while the random matrix is used for XOR-based diffusion. By embedding the noise-like ciphered text within a host image, a visually secure ciphertext image has been generated. The authors in Khan et al. (2020), presented a chaotic visual selective image encryption scheme. The key for the scheme has been derived from the DNA and plaintext image. The system keyspace is increased by using three different chaotic 1D maps. The original image is divided into blocks of varying sizes. Blocks with correlation coefficients above a predefined threshold are XORed with random matrices. The diffused blocks are then permuted to break the correlation between pixel values. As a final step, the ciphertext is encapsulated in a carrier image to create a visually secure ciphertext image.

**Contribution**

- The enhanced medical image encryption scheme has confusion and diffusion characteristics, making it ideal for the IoT environment.
- This scheme resists classical attacks due to its reliance on plaintext images as keys.
- To avoid attackers' attention, ciphered images are embedded in carrier images to produce visually secure images.

The remaining article is organized as follows: Section 2 discusses the preliminaries; Section 3 outlines the proposed methodology; the result analysis of the proposed work is provided in Section 4. Conclusion is provided in Section 5.

## 2 Preliminaries

### 2.1 Chaotic Chen system

**Figure 1:**
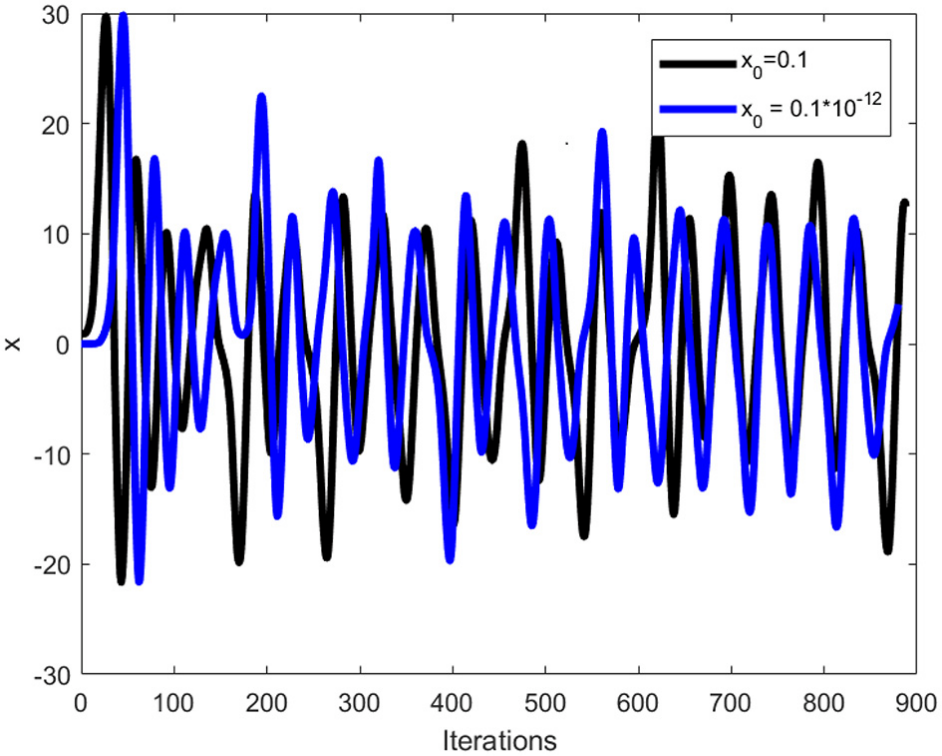
Sensitivity plot of chaotic Chen system.

**Figure 2:**
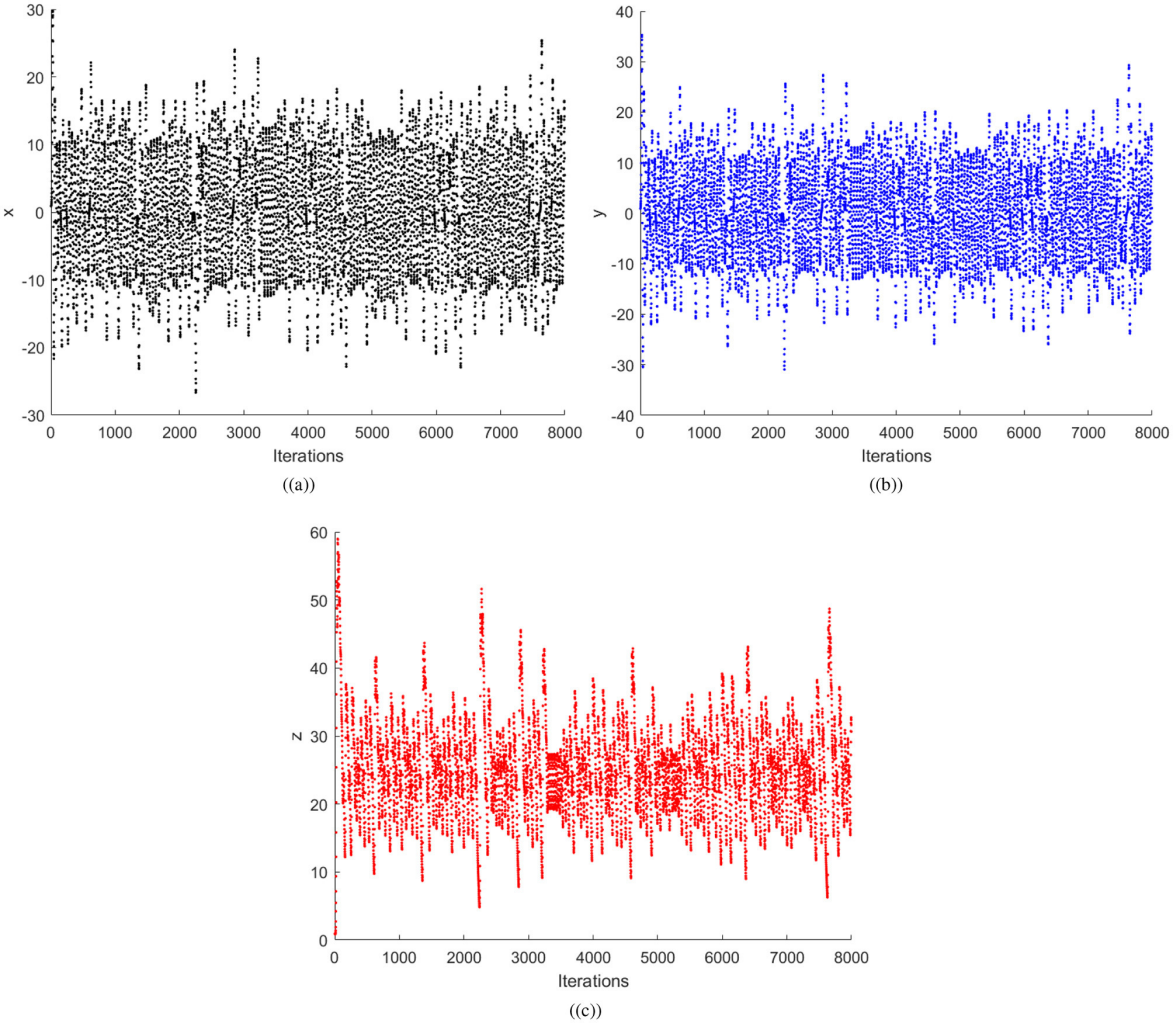
Random number plots: **(a)** x vector, **(b)** y vector, and **(c)** z vector.

Using simple state feedback, Chen developed a new 3D chaotic system in 1999 [1]. Similarly to the Lorenz system, Chen's second and third equations contain cross-product terms. From a topological point of view, the Lorenz and Chen systems have different structures. Mathematically, the system can be written as Qi et al. (2005):


(1) $$\begin{array}{r} \begin{array}{c} ẋ=a*\left(y-x\right),\\ ẏ=\left(\left(c-a\right)*x\right)+\left(c*y\right)-\left(x*z\right),\\ ż=\left(x*y\right)-\left(b*z\right). \end{array} \end{array}$$

where *x, y*, and *z* are the variables indicating the state of the system, and *a, b*, and *c* are the parameters. It has been proven that the Chen system has chaotic behavior for parameter values being α > 0.82 and *a* = 35, *b* = 3, and *c* = 28. In the proposed scheme, the random numbers will be computed using the α = 0.9 value. In order to illustrate Chen system sensitivity, the chaotic system is iterated twice with *x*_0_ = 0.01 and $x_{0}=0.01\times 10^{-12}$. Thus, one can confirms that both the sets of random numbers in Figure 1 are different. Further, Figure 2 shows three sets of 8,000 random numbers generated through the Chen chaotic system. Therefore, one can conclude that the chaotic system is extremely sensitive and produces different random numbers with small changes in the initial condition or control parameter.

### 2.2 SHA-512

In 2002, the National Security Agency (NSA) developed a cryptographic hashing algorithm named Secure Hash Algorithm 2 (SHA-2) (Wang et al., 2021). Compared to its predecessors SHA-0 and SHA-1, SHA-2 provides a more robust solution. SHA-512 is the most secure and efficient hash function in the SHA-2 family (Bhonge et al., 2020). Based on an arbitrary message length, it computes a 512-bit hash value by splitting the data into blocks of 1024 bits and passing the data through the module, consisting of 80 rounds. In our proposed scheme, SHA-512 is used to generate eight 512-bit hash values for eight plaintext bit planes, respectively. The hash values are used to generate the initial conditions of the chaotic system.

## 3 Proposed methodology

**Figure 3:**
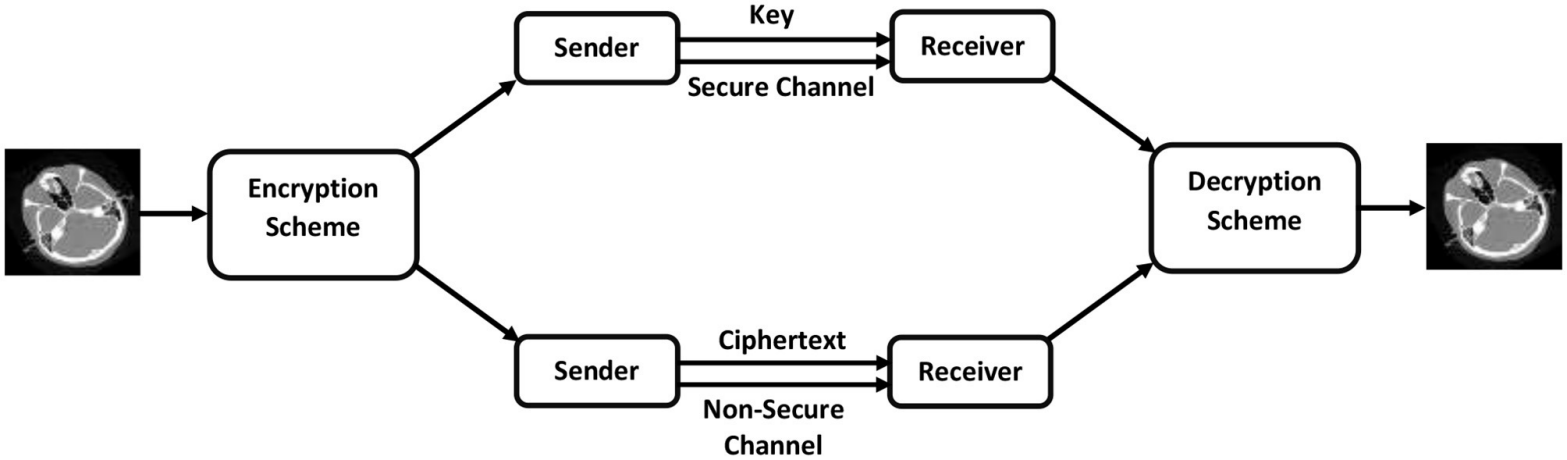
General encryption methodology.

**Figure 4:**
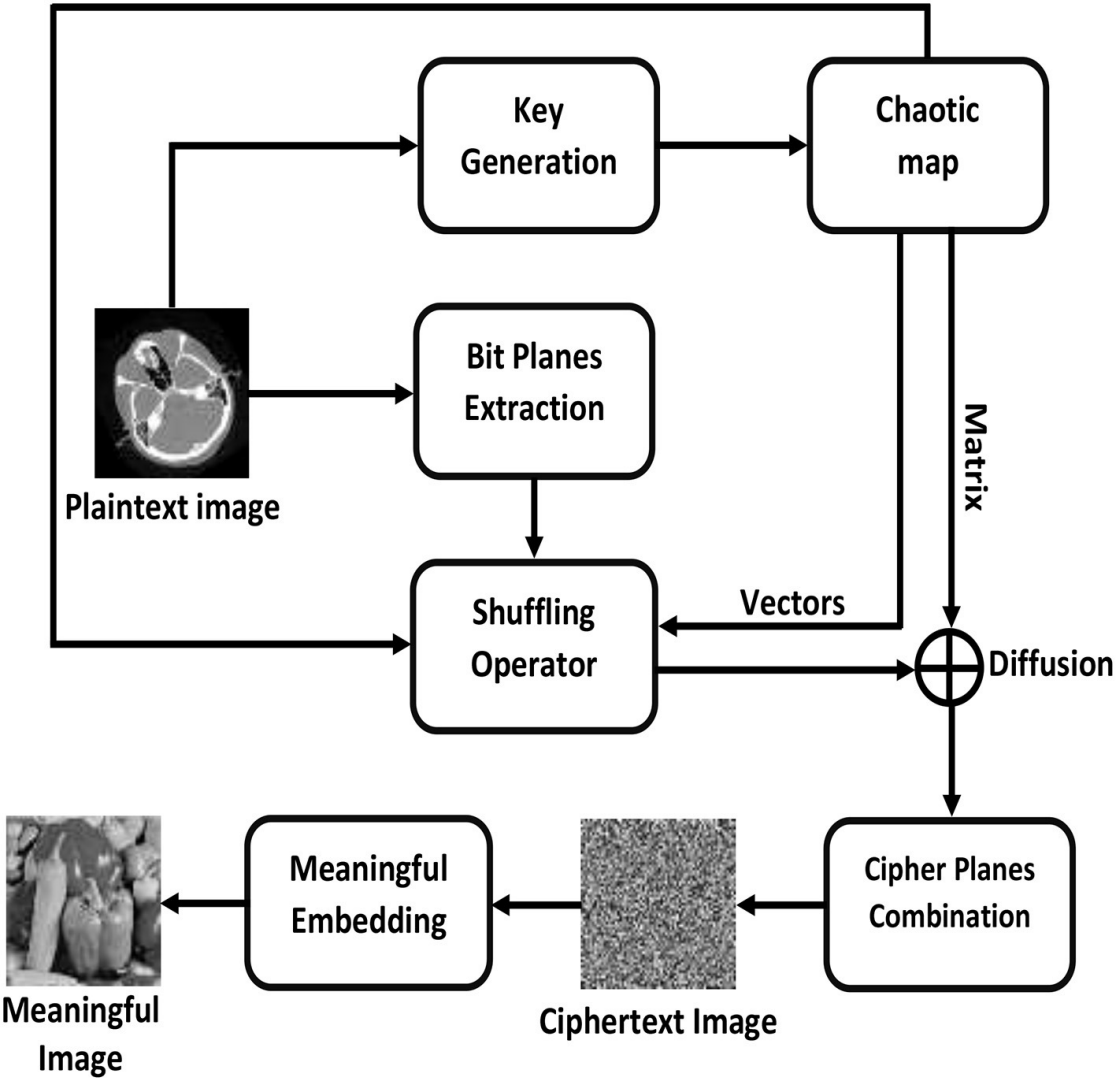
A detailed step-wise flowchart of the proposed scheme.

To divert the attention of an attacker, visually secure encryption facilitates the transfer of private information over an insecure channel. This process embeds the ciphertext image into a carrier or host image to produce visually secure ciphertext images. Figure 3 illustrates the general workflow of an image encryption scheme while Figure 4 demonstrates the step-by-step flow chart of the proposed meaningful privacy preservation of medical images in IoT environments. An end-to-end encryption method has been developed that enables medical images to be transmitted over the Internet using any H-IoT device with enhanced security and confidentiality. The proposed scheme is comprised of the following steps:

**Step: 1** Let the original plaintext medical image with dimensions *m* × *n* can be represented as *M* and its constituent bit planes can be represented as:


(2) $$\begin{array}{l} M=\left[M_{1},M_{2},M_{3},...,M_{8}\right]. \end{array}$$

**Step: 2** To determine the initial conditions for the Chen chaotic system and to ensure the integrity and non-repudiation of the image data, each of these planes is cryptographically hashed utilizing SHA-512.


(3) $$\begin{array}{l} H_{1}=SHA-512\left(M_{1}\right). \end{array}$$

**Step: 3** For numerical interpretation purposes, the computed *H*_1_ value is converted to a decimal value.


(4) $$\begin{array}{l} N=bi2de\left(H_{1}\right). \end{array}$$

**Step: 4** Now, the initial conditions can be calculated as follows:


(5) $$\begin{array}{l} x_{0},y_{0}\;and\;z_{0}=\frac{N}{2^{48}}. \end{array}$$

**Step: 5** The chaotic Chen system is iterated to generate three random vectors *x, y*, and *z*.

**Step: 6** For each generated random vector *x, y*, and *z*, the *Mod*256 is applied to bring the values within the range of 0 and 255.


(6) $$\begin{array}{l} x,y\;and\;z=\text{mod}\left(\left(x,y,\;and\;z\right)\times 10^{14},256\right). \end{array}$$

**Step: 7** The vectors *x* and *y* are utilized to permute the plaintext medical image *M* row- and column-wise, respectively.


$$\begin{array}{l} R_{M}=x\left(M\right), \end{array}$$

(7) $$\begin{array}{l} C_{M}=y\left(R_{M}\right). \end{array}$$

**Step: 8** The vector *z* is rearranged in a matrix and XORed bitwise with the permuted image to generate the final medical bit-plane ciphertext.


(8) $$\begin{array}{l} C_{M}=z⊕C_{M}. \end{array}$$

**Step: 9** Steps 2 through 8 must be repeated eight times to encrypt each layer.

**Step: 10** Combine all ciphertext planes to produce the final ciphertext or encrypted medical image.


(9) $$\begin{array}{l} C=\left[C_{M1},C_{M2},C_{M3},...,C_{M8}\right]. \end{array}$$

**Step: 11** The carrier image *C*_*C*_ is passed through the Lifting Wavelet Transformation (LWT).


(10) $$\begin{array}{l} \left[LL,LH,HL,HH\right]=LWT\left(C_{C}\right) \end{array}$$

**Step: 12** The ciphertext image *C* is divided into 4 Most Significant Bits (MSBs) and 4 Least Significant Bits (LSBs). Now, the *HL* and *HH* blocks of *C*_*C*_ are replaced by the MSBs and LSBs. Finally, the Inverse Lifting Wavelet Transformation (ILWT) was used to generate a visually meaningful medical image *V*_*M*_. As the final visually meaningful medical image *V*_*M*_ contains values greater than 255 and less than 0, it is scaled by a min-max normalization function to keep them between 0 and 255.


(11) $$\begin{array}{l} V_{M}=ILWT\left[LL,LH,MSBs,LSBs\right] \end{array}$$

Decryption can be accomplished by reversing all of the above steps in reverse order.

## 4 Results

**Figure 5:**
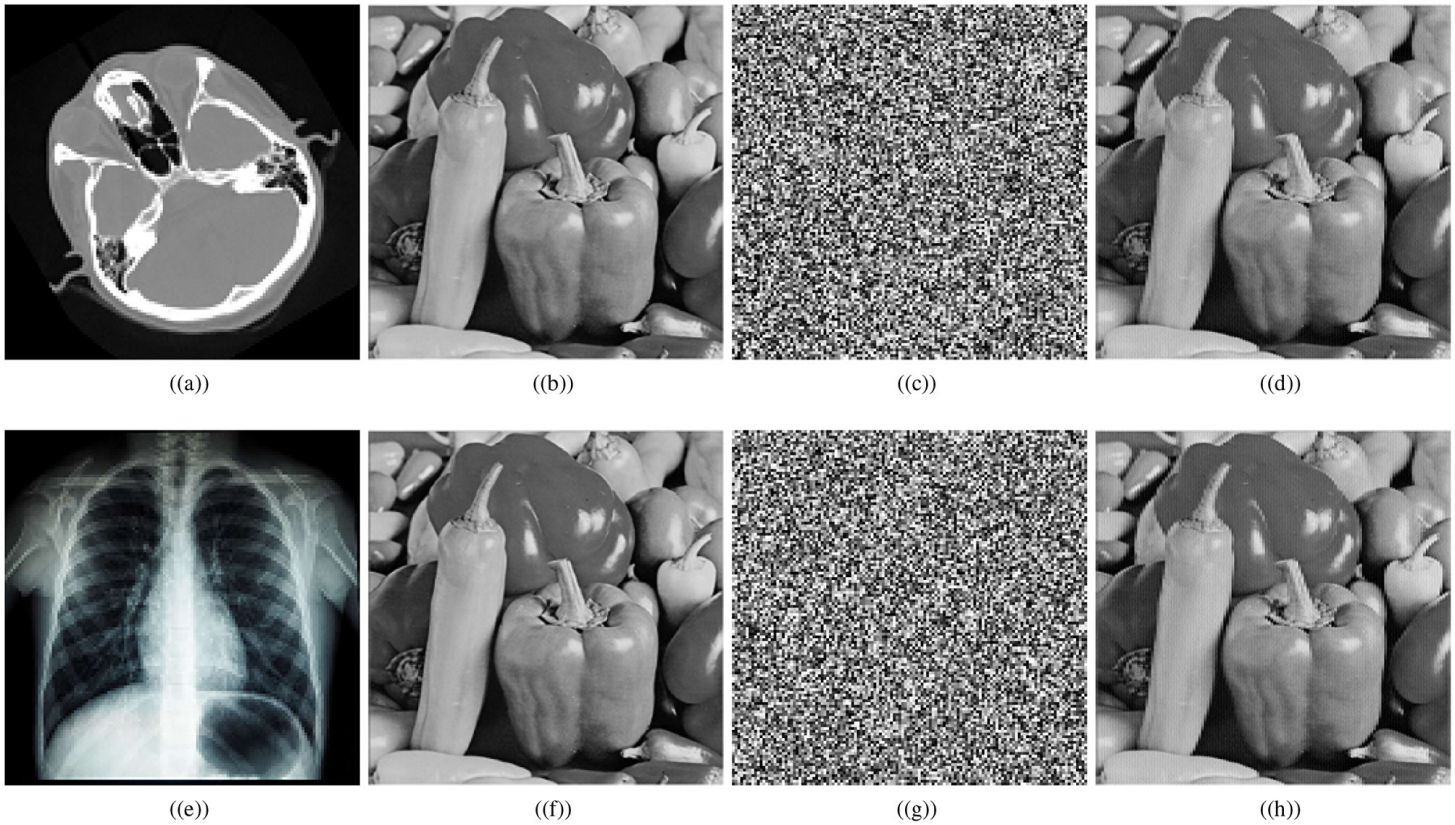
Encryption results: **(a)** plaintext cthead image, **(b)** carrier plaintext image, **(c)** ciphertext cthead image, **(d)** visually secure image, **(e)** plaintext chest image, **(f)** carrier plaintext image, **(g)** ciphertext chest image, **(h)** visually secure image.

This section presents simulations to illustrate the effectiveness and robustness of the proposed scheme. Our analysis in this section demonstrates that the IoT encryption scheme developed for medical images is robust against different security attacks. Figure 5 shows the encryption outcomes of the designed scheme for cthead and chest images of size 128 × 128. The ciphered images in Figures 5c, g are noise-like images, so they are encapsulated inside a carrier image (Pepper image of size 256 × 256) to generate a visually secure medical image. Further, correlation analyses, histogram analyses, entropy analyses, key sensitivity, key space analyses, robustness analyses, etc, are performed to demonstrate the strength of the developed medical image encryption scheme for IoT against statistical attacks, brute force attacks, noise attacks, and classical attacks.

### 4.1 Correlation analysis

Correlation analysis quantifies the relationship between image pixel values. Original plaintext medical images show a close association between neighboring pixels. An encrypted image is secured against pixel relation analysis attacks or statistical attacks when effective cryptographic techniques are applied to reduce the relationship between pixels. A ciphertext image with a lower correlation between adjacent pixels shows a better cryptographic technique. Mathematically, the correlation coefficient can be calculated as follows (Khan and Ahmad, 2019):


(12) $$\begin{array}{l} C.C\left(x,y\right)=\frac{\frac{1}{N}\sum_{n=1}^{N}\left(x_{n}-E\left(x\right)\right)\left(y_{n}-E\left(y\right)\right)}{\sigma_{x}\times \sigma_{y}} \end{array}$$

where


$$\begin{array}{l} \sigma_{x}=\sqrt{Var\left(x\right)} \end{array}$$

$$\begin{array}{l} \sigma_{y}=\sqrt{Var\left(y\right)} \end{array}$$

$$\begin{array}{l} Var\left(x\right)=\frac{1}{N}\overset{N}{\underset{n=1}{\sum }}{\left(x_{n}-E\left(x\right)\right)}^{2} \end{array}$$

$$\begin{array}{l} Var\left(y\right)=\frac{1}{N}\overset{N}{\underset{n=1}{\sum }}{\left(y_{n}-E\left(y\right)\right)}^{2} \end{array}$$

**Figure 6:**
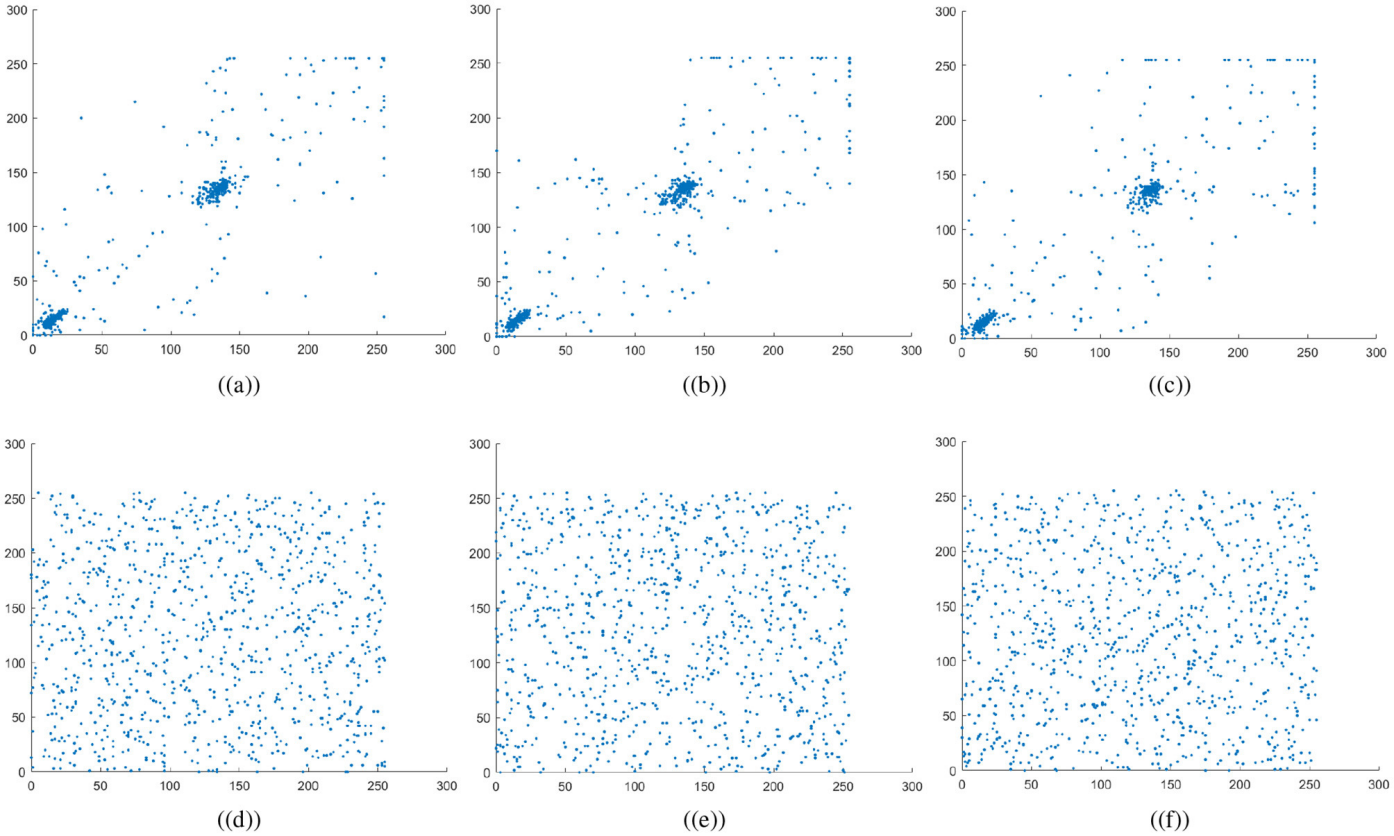
Correlation plots for cthead image: **(a–c)** horizontal direction, vertical direction, and diagonal direction plots for plaintext image, **(d–f)** horizontal direction, vertical direction, and diagonal direction plots for encrypted image.

**Figure 7:**
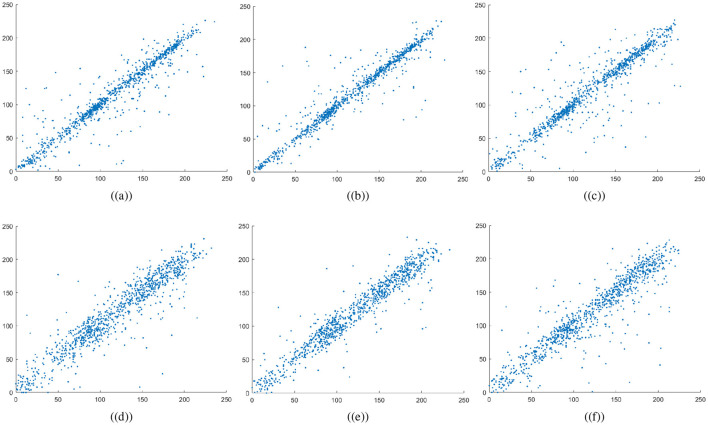
Correlation plots for carrier image: first row are horizontal direction, vertical direction, and diagonal direction plots for plaintext carrier image, while the second row are horizontal direction, vertical direction, and diagonal direction plots for encrypted image.

The variables *N* indicate the total number of pixels while *Var*, σ, and *E* calculate the variance, standard deviation, and expected operator, respectively. Table 1 summarizes the computed correlation coefficient values for the proposed medical image encryption scheme. Almost all the encrypted images have a *C*.*C* value of zero or less than 0. Meanwhile, the carrier or host image and the visually secure image have *C*.*C* values near 1. Thus, embedding the ciphertext medical image does not significantly alter the carrier image. Figures 6a–c illustrates the 5,000 adjacent pixels correlation distribution of the original plaintext cthead medical image in three distinct directions, i.e., horizontal (h), vertical (v), and diagonal (d). Figures 6d–f shows the 5,000 adjacent pixels correlation distribution of the corresponding ciphertext image. Therefore, it can be concluded from Figures 6a–c that neighboring pixels are closely associated in the original plaintext medical image. Furthermore, Figures 6d–f confirms that this association breaks down within the ciphered image's pixels, and the correlation among the pixels is totally different. Additionally, Figure 7 shows a strong association between neighbors pixels in the carrier image and the visually secured image, indicating that the visually secured image's pixels are not significantly changed.

**Table 1:** Computed correlation coefficient values.

**Image**	**Direction**	**Plaintext**	**Ciphertext**	**Carrier**	**Visualy secure**
Cthead	h	0.9480	0.0097	0.9472	0.9343
	v	0.9577	-0.0062	0.9594	0.9585
	d	0.9224	-0.0499	0.9297	0.9227
Chest	h	0.9768	-0.0384	0.9472	0.9368
	v	0.9628	–0.0258	0.9594	0.9368
	d	0.9340	–0.0380	0.9297	0.9055
Medani et al. (2025)	h	0.9173	–0.0598	-	-
	v	0.8868	0.0386	-	-
	d	0.7851	0.0239	-	-
Kumar and Sharma (2024)	h	0.7586	–0.0075	-	-
	v	0.8665	–0.0071	-	-
	d	0.7261	0.0041	-	-

### 4.2 Histogram analysis

**Figure 8:**
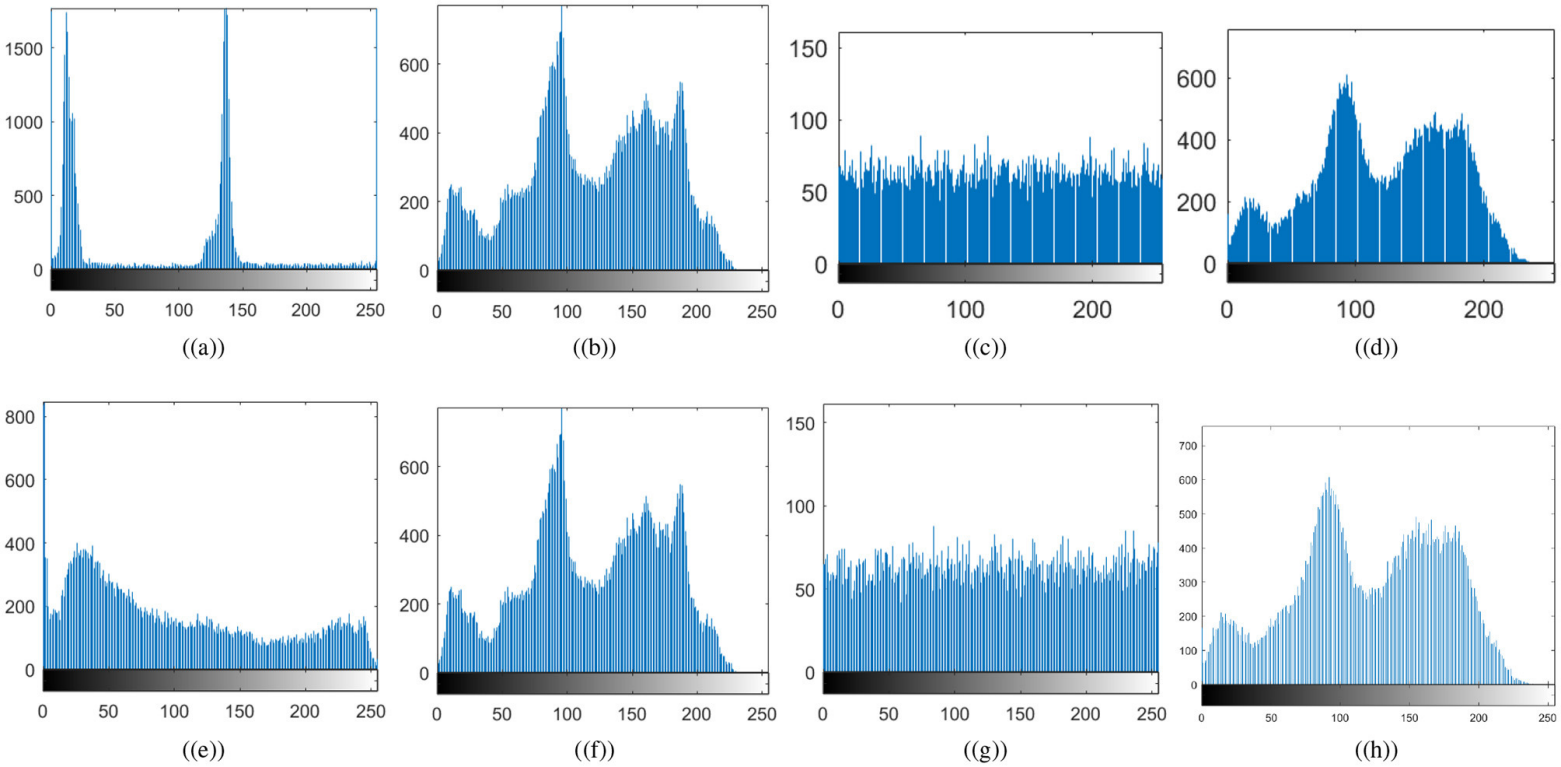
Histogram results: **(a)** plaintext cthead image, **(b)** carrier plaintext image, **(c)** ciphertext cthead image, **(d)** visually secure image, **(e)** plaintext chest image, **(f)** carrier plaintext image, **(g)** ciphertext chest image, **(h)** visually secure image.

Image histograms are statistical plots, plotting the intensity of pixels against the pixel count in a digital image. Mathematically, it can be computed as follows (Singh and Kumar, 2025).


(13) $$\begin{array}{l} H\left(x_{i}\right)=m_{i} \end{array}$$

where *m*_*i*_ represents the multiplicity of *x*_*i*_ intensity number. Histogram analysis helps to determine whether pixel intensities are distributed evenly throughout the encryption process. An encryption scheme's robustness against statistical attack can be assessed by ensuring that the encrypted image's histogram is uniform, making it impossible to use statistical analysis to guess the original image's structure (Khan and Ahmad, 2019). Figure 8 shows the histograms of the original and cipher images. Figure 8 confirms the non-uniformity of the histograms for the original cthead and chest images; that is, some pixel intensities may be dominant depending on the contents of the image. In contrast, the cipher images' histograms are uniformly distributed. As a result, the encryption process scrambles pixel values such that no feature of the plaintext image can be identified. Because of the histogram's uniformity, the proposed medical image encryption for IoT is highly resistant to statistical attacks. Histograms of carrier images and visually secured images appear to be nearly identical. Thus, the attacker will not be able to determine that the carrier image is embedded with an encrypted image, as the embedding is not producing significant changes in the host image.

### 4.3 Key space

In an encryption algorithm, key space refers to all possible secret keys and different parameters. The authors in Alvarez and Li (2006) illustrate how key space size influences the strength of image ciphering techniques. It is essential that the key space be sufficiently large and must exceed 2^100^ to withstand brute force attacks. The proposed meaningful privacy preserving of medical images in IoT environment utilizes the Chen chaotic system, with state variables *x, y*, and *z* and control parameters *a, b*, and *c*. Each of these parameters has a floating precision of 10^15^. Further, the map is iterated 8 times for each bit plane. Therefore, the key space of the designed scheme can be computed as follows:


$$\begin{array}{l} K={\left(10^{15}\times 10^{15}\times 10^{15}\times 10^{15}\times 10^{15}\times 10^{15}\right)}^{8} \end{array}$$

(14) $$\begin{array}{l} K={\left(10^{90}\right)}^{8}>>2^{100} \end{array}$$

Furthermore, the key space computed in Kanwal et al. (2024) and Medani et al. (2025) is 2^282^ and 2^598^, respectively. Thus, one can conclude that the key space of the presented medical image encryption scheme is sufficiently large to resist a brute force attack significantly.

### 4.4 Key sensitivity

**Figure 9:**
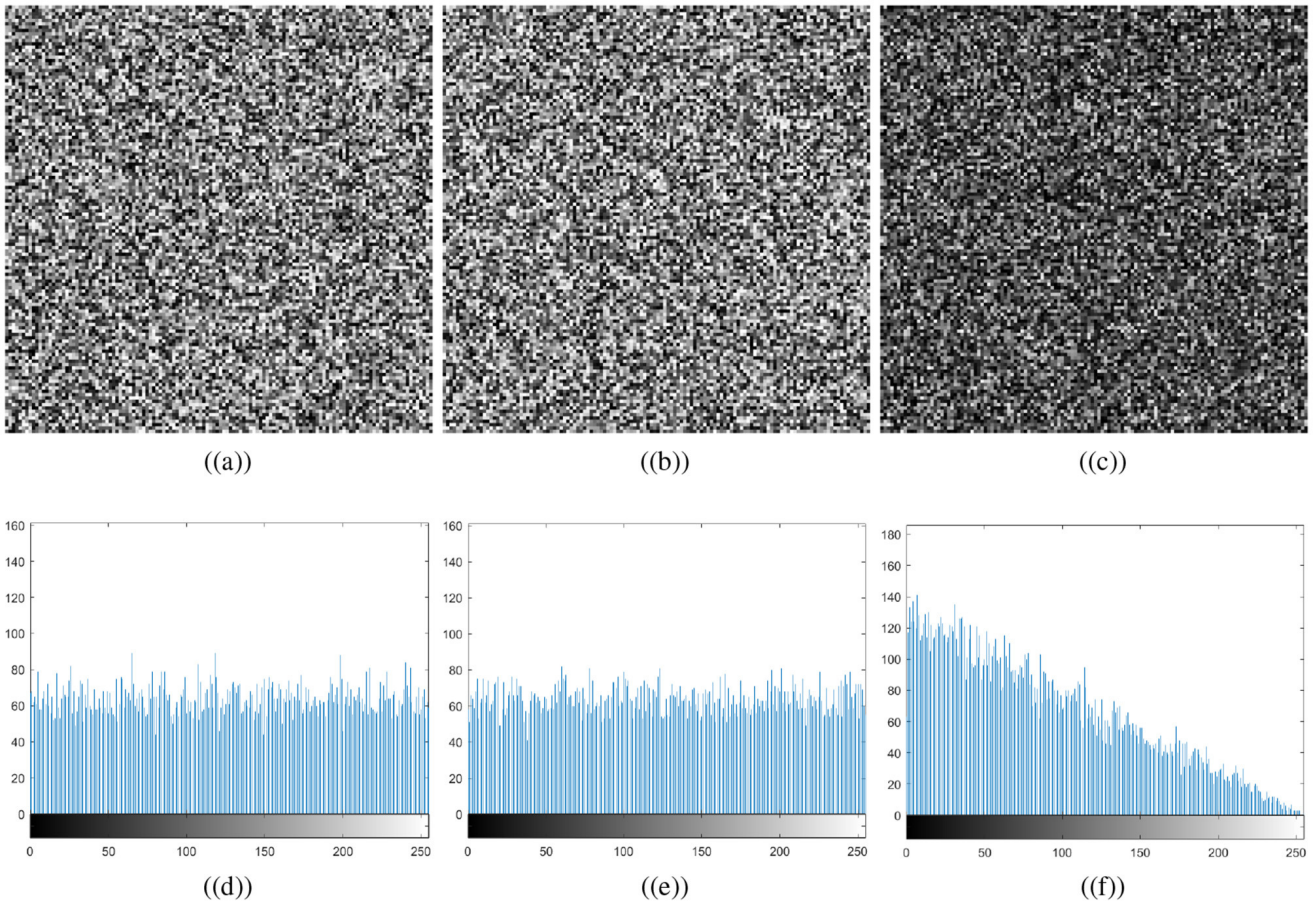
Key sensitivity plots: **(a)** original key, **(b)** modified key, **(c)** differential image, and **(d–f)** are the corresponding histograms.

A good image encryption technique should be able to detect subtle changes in secret keys and parameters, resulting in decoded data that is different from plain image data. The proposed medial image encryption is extremely sensitive to the control parameters and initial conditions. Let's make a small change of 10^−12^ in one of the initial conditions or the control parameters, i.e., *x*_0_ of the Chen chaotic system. As a result, the chaotic system will generate different random numbers. Figure 1 shows the different number generation for a small modification in the initial conditions. Figure 9 illustrates the resultant images after decrypting the ciphertext cthead image with the same and modified keys. A differential image of the two resultant images is shown in Figure 9c. A small change to the initial conditions or control parameters of the Chen chaotic system will fail the decryption process, resulting in a completely different image for the attacker. The differential image demonstrates that both resultant images are different and lack any recognizable information related to the plaintext cthead image. It can therefore be concluded that the proposed meaningful medical image encryption scheme is exceptionally sensitive to even minor changes in the chaotic system control parameters.

### 4.5 Entropy analysis

Entropy analysis is usually used to assess image encryption's robustness against entropy attacks. Mathematically, entropy of a data source can be computed as follows (Singh and Kumar, 2025):


(15) $$\begin{array}{l} E\left(S\right)=\overset{256-1}{\underset{k=0}{\sum }}\left(\frac{1}{256}\right)log_{2}\left(\frac{1}{\frac{1}{256}}\right) \end{array}$$

To resist the entropy attack, the entropy value of the encrypted images should be close to 8. Table 2 summarizes the computed entropy values for the proposed medical image encryption scheme. Thus, one can confirm that the entropy value of the ciphertext medical image is approximately equal to 8. The designed technique is robust against entropy attacks without exposing sensitive information.

**Table 2:** Computed entropy values.

**Image**	**Plaintext**	**Ciphertext**	**Carrier**	**Visualy secure**
Cthead	5.6763	7.9987	7.6110	7.6485
Chest	7.4040	7.9982	7.6110	7.6498
Medani et al. (2025)	7.6414	7.9998	-	-
Kumar and Sharma (2024)	7.3579	7.9987	-	-

### 4.6 Differential attack analysis

To measure the effectiveness and reliability of image encryption algorithms against differential attacks, it is important to determine the Number of Pixels Change Rate (NPCR) as well as the Unified Average Change Intensity (UACI). These two matrices can be mathematically defined as follows (Liu et al., 2024):


(16) $$\begin{array}{c} D\left(x,y\right)=\{\begin{array}{c} 1,ifC_{1}\left(x,y\right)\neq C_{2}\left(x,y\right)\\ 0,ifC_{1}\left(x,y\right)=C_{2}\left(x,y\right) \end{array} \end{array}$$

(17) $$\begin{array}{c} NPCR=\underset{x,y}{\sum }\frac{D\left(x,y\right)}{N}\times 100\% \end{array}$$

(18) $$\begin{array}{c} UACI=\frac{1}{N}\underset{x,y}{\sum }\frac{\left|C_{1}\left(x,y\right)-C_{2}\left(x,y\right)\right|}{255}100 \end{array}$$

where *N* shows the total number of pixel values and *C*_1_ represents the first encrypted image generated without any change in the original plaintext image while *C*_2_ represents the encrypted image generated after altering just one pixel in the original image. When comparing two images that have been encrypted, the UACI test measures the difference in pixel intensity, whereas the NPCR test measures how frequently the pixels are changed in the plaintext. The calculated NPCR and UACI values for the designed medical image security scheme are illustrated in Table 3. Therefore, the values UACI > 33% and NPCR > 99% confirm that the proposed strategy is resilient to differential attack.

**Table 3:** Number of pixels change rate and unified average change intensity computed values.

**Image**	**NPCR**	**UACI**
Cthead	99.6755%	33.5105%
Chest	99.6867%	33.5241%
Medani et al. (2025)	99.6653%	33.5328%
Kumar and Sharma (2024)	99.5800%	33.1800%

### 4.7 Noise attack analysis

**Figure 10:**
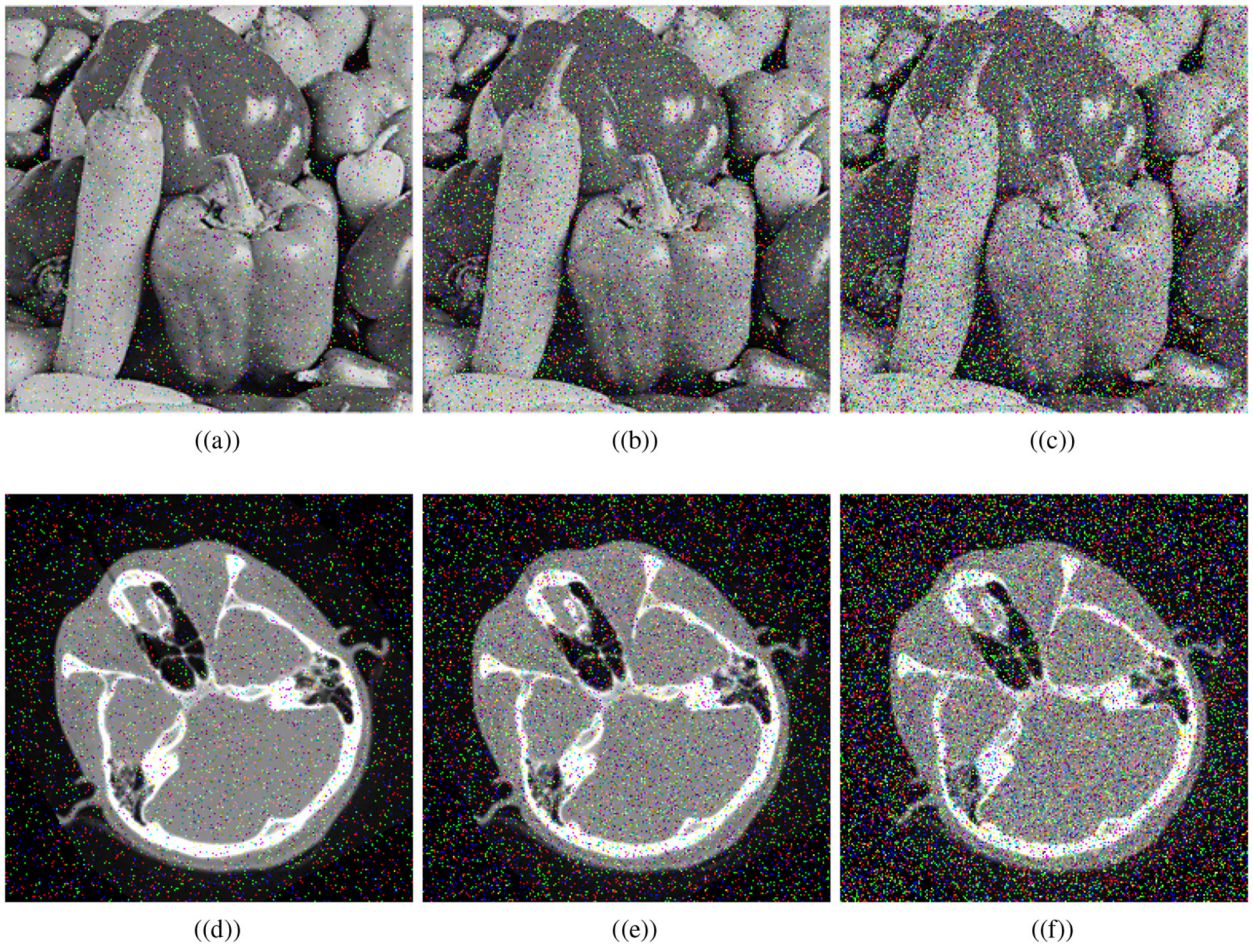
Noise results: salt and pepper noise ratios; **(a)** 5%, **(b)** 10%, **(c)** 20%, and **(d–f)** corresponding decrypted images.

It has become increasingly important to analyze noise attacks when data is transmitted over open networks due to the presence of noise during transmission. Therefore, the proposed algorithm's effectiveness is determined by comparing the decryption of encrypted images under different noise intensities. Figure 10 shows the recovered images after adding salt and pepper noise of (5%, 10%, and 20%) intensities to the visually secured image. Thus, one can see that the proposed medical encryption scheme can decrypt the noise-polluted ciphertext image, illustrating the robustness of the scheme.

### 4.8 Occlusion attack analysis

**Figure 11:**
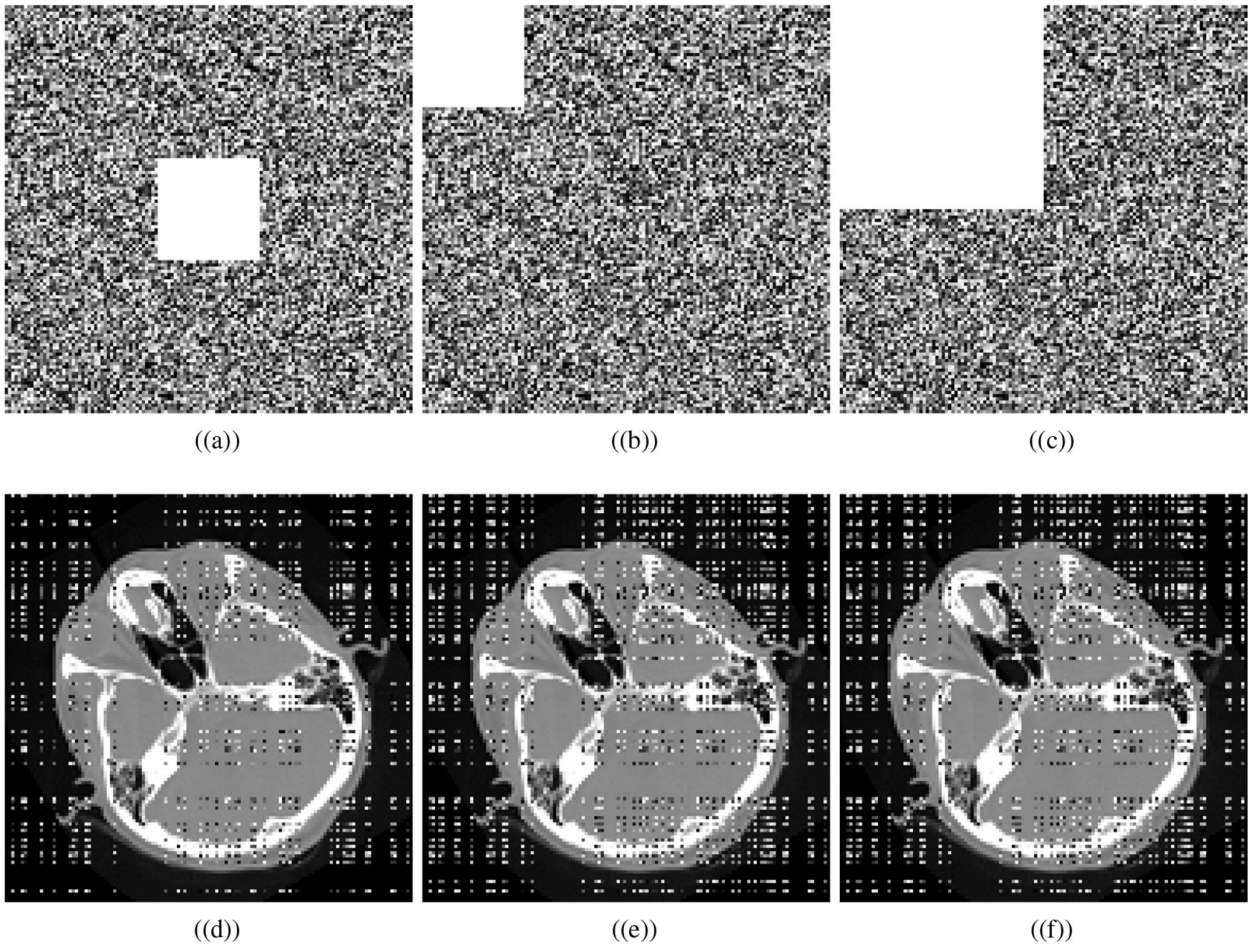
Cropping results: Crop ratios of ciphertext cthead image; **(a)** 1/16 (middle), **(b)** 1/16, **(c)** 1/4, and **(d–f)** corresponding decrypted images.

**Figure 12:**
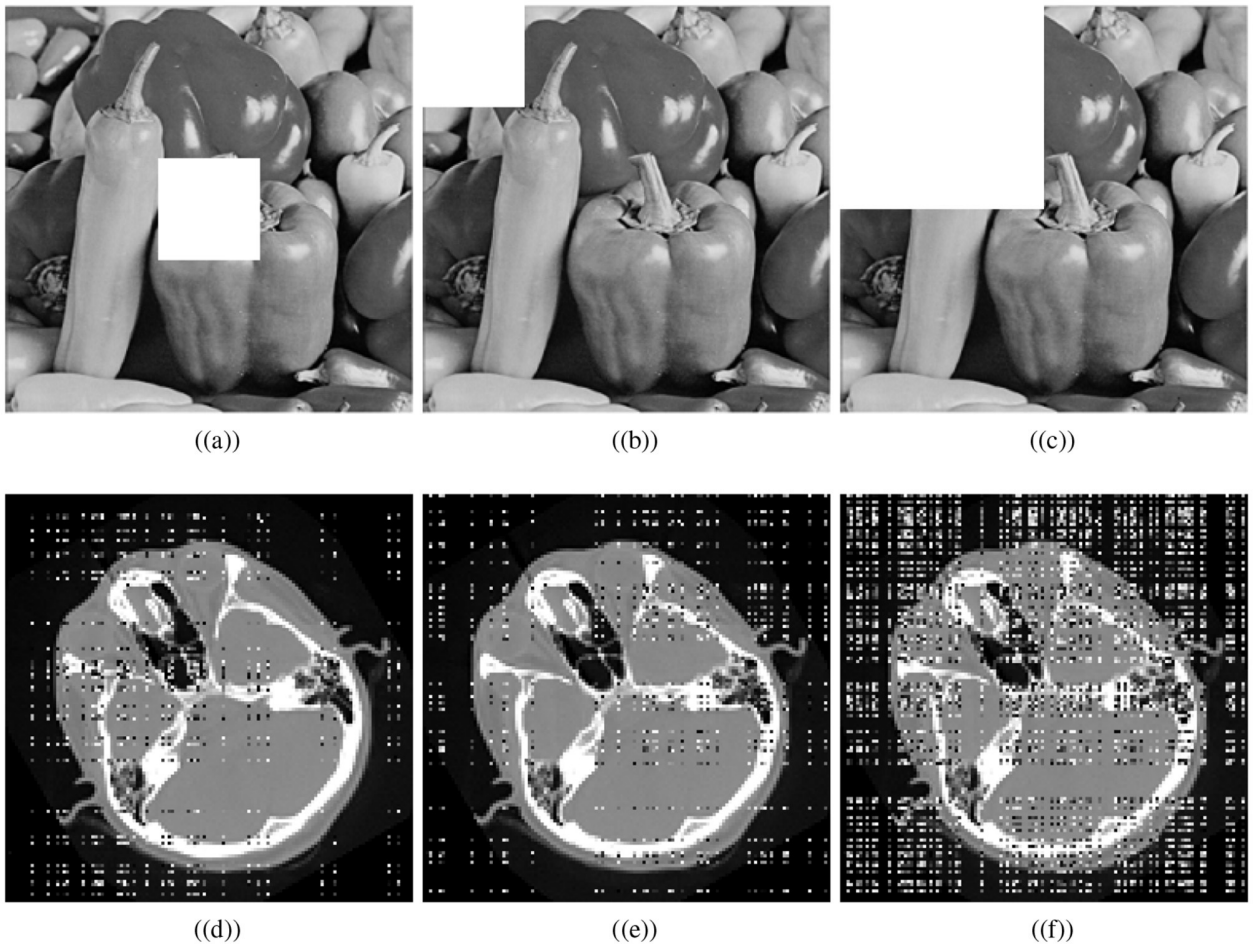
Cropping results: Crop ratios of visually meaningful image; **(a)** 1/16 (middle), **(b)** 1/16, **(c)** 1/4, and **(d–f)** corresponding decrypted images.

Various factors can cause data to be lost during image transmission over a network. The purpose of occlusion analysis is to determine whether or not an image encryption scheme can recover a plaintext image from a ciphertext image that has been occluded. Different-sized portions of the encrypted image are cropped and decrypted. This analysis can provide insight into how the encryption scheme scrambles plaintext images. Generally, the better the scrambling effect, the more likely the algorithm is to reconstruct the visual characteristics of the plaintext image, even if some part of it has been lost. We cropped the cipher cthead image and visually secured image with the ratios 1/16 (middle), 1/16, and 1/4. Decryption is performed utilizing the presented scheme. Figure 11 shows the cropped ciphertext images and the corresponding decipher images while Figure 12 illustrates the cropped visually secured images and the corresponding decipher images. The visual results clearly deomnstrates that the proposed scheme strongly deciphers the cropped images without causing any noticeable distortion.

### 4.9 Resilience against classical attacks

Classical attack analysis focuses on identifying and analyzing various types of attacks (known plaintext, chosen plaintext, ciphertext only, and chosen ciphertext) against encrypted images. The key of the chaotic maps is computed based on the plaintext hash value. It is used to determine initial conditions and control parameters. Because of the dependence on plaintext images, the proposed enhanced medical image privacy in IoT with bit-plane level encryption using a chaotic map avoids the classical attacks cited above. Therefore, all random vectors and matrices are determined by plain image bit planes. When a single pixel is changed in the plaintext image, the keystream changes. This will result in a completely different ciphertext image.

### 4.10 Complexity analysis

Time complexity is a metric used to estimate the running time of an encryption algorithm and generally determine the scheme's feasibility. A good algorithm needs to have a short running time. The encryption and decryption results are performed on MATLAB 2018a with Microsoft Windows 10, 4 GB of memory, and a 1 GHz CPU. The cthead and chest images have a size of 128 × 128, while the carrier image has a size of 256 × 256. The proposed scheme takes 0.85s to generate the ciphertext image and 0.62s to produce the visually meaningful ciphertext. Thus, the proposed scheme takes 1.47s to generate the final meaningful ciphertext. The image encryption scheme presented in Kumar and Sharma (2024) takes 0.85s while the scheme discussed in Medani et al. (2025) takes 4.57s to produce the final encrypted images. The designed scheme takes less time than the scheme presented in Medani et al. (2025) and more time than the scheme discussed in Kumar and Sharma (2024).

## 5 Conclusion

This paper presents a novel and robust medical image encryption scheme for resource-constrained devices. Due to simplicity and exceptional performance in terms of unpredictability, the proposed scheme utilizes 3D Chen chaotic system. The simplicity and excellent performance make the Chen chaotic map an excellent choice for lightweight encryption applications. The designed meaningful bit-plane-level medical image encryption scheme for IoT leverages the pixel scrambling and diffusion characteristics to effectively break pixel relationships, thus, enhancing encryption efficiency and security. To enhance security, the plaintext bit planes are hashed using the Secure Hash Algorithm (SHA-512) to compute the initial conditions of the chaotic map. This dependency on the plaintext images makes the designed scheme resilient against classical attacks (known-plaintext, chosen-plaintext, ciphertext-only, and chosen-ciphertext). As a result, three random vectors for permutation and XOR diffusion are generated. A permutation and XOR operation are applied to each bit-plane to produce a ciphertext plane. After combining the ciphertext bit-planes, the visually secured ciphertext image is now generated by embedding the ciphered image within the carrier image. Extensive evaluations have proven that the designed scheme exhibits a high degree of resilience to attacks, making it particularly suitable for small IoT devices with limited processing power and memory. Computational complexity could be a possible limitation of the designed scheme, as image sizes increase, the encryption process could take longer.

## Data Availability

Interested researchers may contact the author for potential collaboration or for inquiries regarding data access within the constraints of institutional guidelines.
